# Clinical microbiology in detection and identification of emerging microbial pathogens: past, present and future

**DOI:** 10.1080/22221751.2022.2125345

**Published:** 2022-11-04

**Authors:** Hui Wang, Wenhong Zhang, Yi-Wei Tang

**Affiliations:** a Department of Clinical Laboratories, Peking University People's Hospital, Beijing, People’s Republic of China; b Department of Infectious Diseases, National Medical Center for Infectious Diseases, Huashan Hospital, Fudan University, Shanghai, People’s Republic of China; c Medical Affairs, Danaher Diagnostic Platform China/Cepheid, Shanghai, People’s Republic of China

**Keywords:** Clinical microbiology, nucleic acid amplification, genomics, transcriptomics, proteomics, metabolomics, point of care, COVID-19

## Abstract

Clinical microbiology has possessed a marvellous past, an important present and a bright future. Western medicine modernization started with the discovery of bacterial pathogens, and from then, clinical bacteriology became a cornerstone of diagnostics. Today, clinical microbiology uses standard techniques including Gram stain morphology, *in vitro* culture, antigen and antibody assays, and molecular biology both to establish a diagnosis and monitor the progression of microbial infections. Clinical microbiology has played a critical role in pathogen detection and characterization for emerging infectious diseases as evidenced by the ongoing COVID-19 pandemic. Revolutionary changes are on the way in clinical microbiology with the application of “-omic” techniques, including transcriptomics and metabolomics, and optimization of clinical practice configurations to improve outcomes of patients with infectious diseases.

The unprecedented outbreak of the coronavirus disease 2019 (COVID-19) pandemic has highlighted the necessity for readily available, accurate and fast diagnostic testing methods to detect and characterize emerging pathogens. The basic work of the clinical microbiology laboratory is to provide evidence for the diagnosis, treatment, and control of infectious diseases by detecting the presence of specific pathogenic microorganisms in clinical specimens. When a pathogen is detected, it is then subjected to a series of further analyses, including identification, typing, quantification, and antimicrobial susceptibility testing [1,2].

## A long and uneven past

The discipline of clinical microbiology has been evolving for more than two hundred years since Dutchman, Anton van Leeuwenhoek invented the microscope (Table 1). The development of the light microscope was the foundational technological advancement in modern medicine for the direct visualization of microorganisms. By the 1830s, Lister introduced the “achromatic” lens to eliminate the blurring and colour distortion known as “chromatic aberration,” which had previously limited resolution at higher magnification (i.e. bacterial level) [3]. These technical advancements in microscopy converged with seminal concepts in bacteriology from the work of Pasteur and Koch. Together, they allowed the microscope to serve as a powerful instrument for physicians and microbiologists to directly visualize pathogens in human specimens, especially with the application of (still universal) techniques such as the Gram and acid-fast staining procedures, also developed in the late nineteenth century [2,4].

**Table 1.** Historical figures in clinical microbiology.

	Scientist	Year of birth and death	Major contributions in clinical microbiology
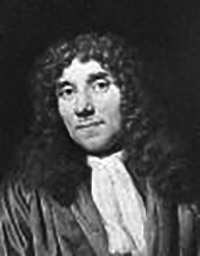	Anton van Leeuwenhoek	1632–1723	Invented microscope to observe microorganisms
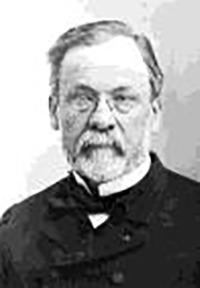	Louis Pasteur	1822–1895	Found microorganisms in infected patients
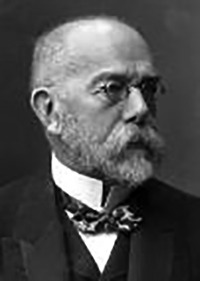	Robert Koch	1843–1910	Established three criteria for determining a causative relationship between a microbe and a disease
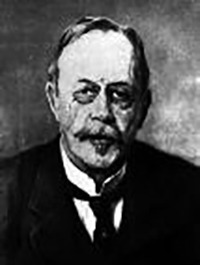	Hans Christian Gram	1853–1938	Invented the Gram stain
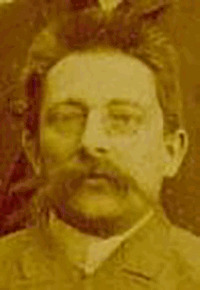	Julius Richard Petri	1852–1921	Invented Petri dishes to start in vitro culture
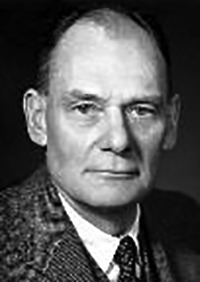	John Franklin Enders	1897–1985	Applied cell culture techniques for isolation and growth of viruses
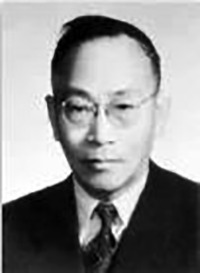	Feifan Tang	1897–1958	Discovered and cultured the pathogen causing trachoma
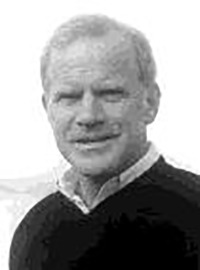	Kary Banks Mullis	1944–2019	Invented polymerase chain reaction for in vitro nucleic acid amplification

Equally important during this period were newfound abilities to culture microorganisms from human sources. While early bacterial cultures were accomplished with slices of raw potato, the 1887 invention of the petri dish facilitated the direct observation of colonies with gelatine or agar, allowing for the morphological description of species in pure culture [5]. It likewise allowed for their biochemical characterization, creating the phenotypic profiles that have served as the basis for taxonomic identification in clinical laboratories until the twenty-first century. Culture is likewise a pre-requisite for phenotypic antimicrobial susceptibility testing (AST), which remains the gold standard for predicting the response of a patient’s bacterial/fungal infection to treatment [1,4,6].

Shortly after the development of axenic bacterial/fungal culture, methods for propagating human viruses using ex vivo cellular substrates, fertilized chicken embryos, and *in vitro* cell lines became available. In the late 1940s, John Franklin Enders first applied cell culture techniques to isolate and grow poliovirus, initiating modern clinical virology [7]. In 1956, Feifan Tang, a Chinese microbiologist and virologist, discovered and isolated *Chlamydia trachomatis*, which clarified the cause of trachoma [8]. For decades, tissue and cell culture-based methods have played critical roles in rapid identification of emerging pathogens and exploration of pathogenesis. This has been demonstrated in three unprecedented outbreaks of emerging human coronavirus (SARS-CoV, MERS-CoV and SARS-CoV-2) infections since the beginning of the twenty-first century [9]. More importantly, cell culture techniques, along with chicken egg embryos, have become the main tools to reproduce large quantities of viruses for vaccine manufacturing for fighting emerging infectious diseases [10].

Alongside these abilities to visualize and cultivate organisms came new techniques that assess the body’s humoral response to infection. This principle was first established in 1892 by Sternberg using the vaccinia virus [11]. It is now recognized that antibodies within serum facilitate identification not just of viruses, but multiple types of pathogens by the clinical laboratory [12,13]. The value of diagnostic serology for infectious diseases is founded on two criteria: (i) antibodies are specific for a particular cellular target and (ii) antibodies are induced by a specific stimulus. Ideally, the definitive evidence for infection by a pathogen would involve recovery of that agent from infected tissue, along with an increase in specific antibodies over time. With some infections, however, the recovery of the causative agent may be difficult, dangerous, impractical, or even impossible [13]. Certainly before the development nucleic acid assays (and in some cases still), the combination of appropriate clinical and serologic findings can serve as sufficient criteria for diagnosis, even if the organism itself is not detected [12]. Seropositivity can likewise function as a marker of functional immunity toward many (but not all) pathogens, and these tests facilitate population monitoring within the fields of epidemiology and public health [12,13]. Moreover, the purification of pathogen-specific antibodies allows for the development of “antigen-based” diagnostics that detect microbe-specific biomolecules other than nucleic acids, typically carbohydrates and proteins.

Kary Banks Mullis, an American biochemist and Nobel Prize winner, was credited with the invention of the polymerase chain reaction (PCR) in the 1980s for nucleic acid amplification, thus applying rapid, sensitive and specific molecular biology techniques to the practice of clinical microbiology [14,15]. A quick application of reverse transcriptase (RT), an enzyme recovered in 1970 simultaneously by Baltimore and Temin [16,17], resulted in RT–PCR which has been widely used in the detection and characterization of RNA targets. Further modification of PCR into a quantitative format (qPCR) by Higuchi et al. in 1993 has enabled accurate determination of pathogen loads in clinical specimens [18]. Additionally, the automation of the Sanger sequencing method by Leroy Hood and Michael Hunkapillar at Applied Biosystems in 1987 rendered the rapid generation of complete genome sequences of *Haemophilus influenzae* [19] and *Mycoplasma genitalium* [20] a reality [21].

## A flourishing present

In the past decades, clinical microbiology laboratories have undergone important changes with the introduction of molecular biology techniques [22] and laboratory automation [23]. Diagnostic methods in clinical microbiology are currently divided into the following five categories (Table 2) [1]. The first one is the morphological observation under the naked eye or microscope. This is a fundamental method, which is currently used mainly for initial screening and for guiding the next step of testing. Specific morphological findings by microscopy can quickly identify the pathogenetic agent in some cases. For example, the finding of Gram-negative diplococci in urethral exudates from males is a reliable indication of *Neisseria gonorrhoeae* infection. The second is the antigen test for pathogenic microorganisms. It is widely used in clinical practice for outpatient testing because of its rapidity, simplicity, and specificity [24,25]. Examples for this include laboratory testing for the urine pneumococcal and *Legionella* antigen tests [26]. On the other hand, the disadvantage of antigen tests lies in its often poor sensitivity. Specimens that are antigen-negative in clinical practice usually need to be retested with more sensitive methods, such as culture or/and molecular methods to avoid a missed diagnosis. Examples for this include laboratory testing for *Streptococcus pyogenes* [24] and influenza virus [25].

**Table 2.** Microbiomic technology main contents. MALDI-TOF MS, matrix assisted laser desorption ionization time of flight mass spectrometry.

	Technology	Target molecule	Question addressed	Output	Main methods	Selected references
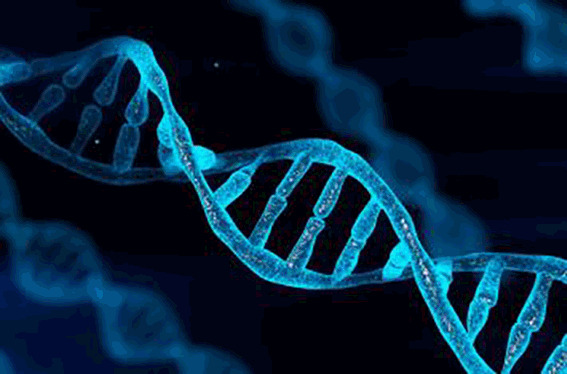	Genomics	DNA	Infection potential	DNA sequences	DNA sequencing	[69,71,72]
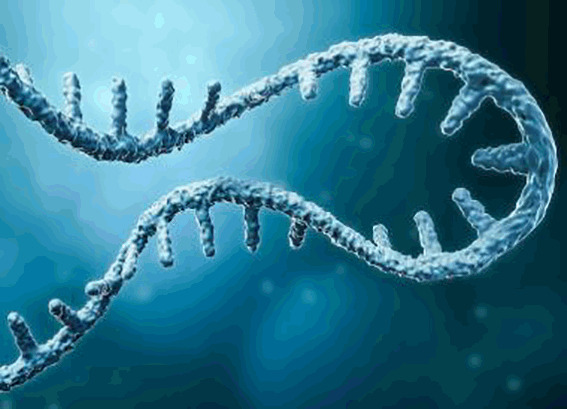	Transcriptomics	RNA	Infection strategy	Transcription of text	RNA sequencing, quantitative transcription PCR	[77,82,84]
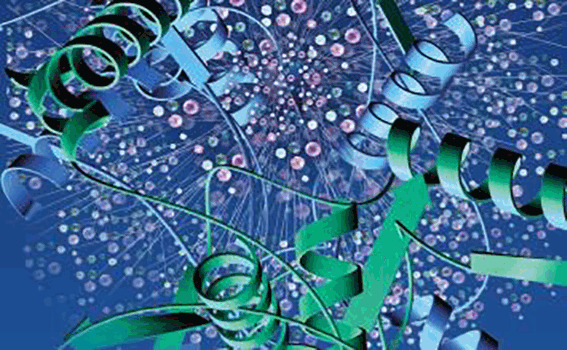	Proteomics	Protein	Infection process	Protein profiles	MALDI-TOF MS	[83,89,90]
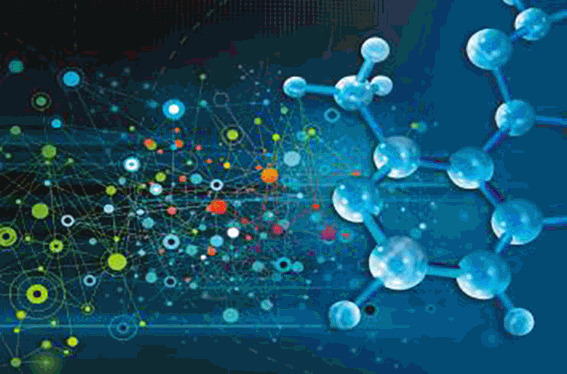	Metabolomics	Metabolites	Infection outcome	Metabolic text	Gas or liquid phase mass spectrometry	[78–80]

During the ongoing COVID-19 pandemic, the performance of rapid antigen tests for COVID-19 diagnosis has been widely evaluated and found to be varied in different settings [27]. Rapid antigen tests are cheaper and provide faster results, thus potentially enabling prompt isolation of positive cases and quarantine of close contacts. A recent literature review covering a total of 16 studies reported on the effectiveness of rapid antigen testing for screening of asymptomatic individuals to limit the transmission [9,28]. Eight included studies examining the effectiveness of rapid antigen testing for population-level screening, four for pre-event screening and four for serial testing. Overall, there was no evidence regarding the effectiveness of rapid antigen testing for the screening of asymptomatic individuals to limit the transmission of SARS-CoV-2. This uncertainty is due to the inconsistent results, the relatively low number of studies identified, the predominantly observational and/or uncontrolled nature of the study designs used, and concerns regarding methodological quality. Given this uncertainty, more real-world research evidence in relevant settings, which is of good quality and timely, as well as economic evaluation, is required to inform public policy on the widespread use of rapid antigen tests in asymptomatic individuals [28].

The third category is the culture method which remains the gold standard for culturable pathogenic microorganisms [1]. It mainly includes inanimate media such as agar or broth and animate media such as tissues and cells. The former is mainly used for culturing extracellular pathogens such as bacteria and fungi, while the latter is mainly used for culturing intracellular pathogens such as viruses/chlamydia. Cultivation for bacteriology and mycology has gradually transitioned into a totally automated continuous monitoring mode of blood culture/liquid culture from the manual method. Viral cultures are still an important research and clinical laboratory tool when the potential viral agent is not known. Other application niches include documenting active infection, performing antiviral susceptibility testing and developing a vaccine and therapeutic agents.

Viral culture has been a valuable tool for studying COVID-19 pathogenesis, resulting in developing more effective disease prevention, diagnosis, and control. To combat COVID-19, since SARS-CoV-2 was first isolated from nasopharyngeal and oropharyngeal specimens from a patient with COVID-19 in Vero-CCL81 and Vero E6 cells [29], several *in vitro* and ex vivo cell culture systems have progressively been used and described [30]. Matsuyama et al engineered a Vero E6 cell line expressing TMPRSS2 for culturing of SARS-CoV-2 showing more than 100 times greater production of viral RNA copies than Vero E6 cells alone [31]. Cell culture remains the definitive assay to determine viral infectivity and transmission. Using cell culture methods, Wölfel et al revealed that infectious viruses were readily isolated from samples derived from the throat or lung, but not from stool samples, in spite of high concentrations of virus RNA. Blood and urine samples never yielded virus [32]. A recent review by Bhat et al summarized that infectious virus was generally not shed beyond 20 days of the onset of symptoms in most COVID-19 patients, including severely ill and immunocompromised, as indicated by failure to isolate replication-competent virus by viral culture [33]. Cell culture-based plaque reduction neutralization assays and derivatives are the most reliable and accurate methods to determine SARS-CoV-2 neutralization antibody activities [34,35].

The fourth category is the serological technique to detect the specific host response to pathogenic microorganisms causing infection. However, due to the lag time to a detectable host response and the cross-reactivity between similar pathogens, serological methods are rarely used for the rapid detection of infections. Successful examples are the detection of pathogen-specific IgM antibodies for hepatitis A virus and perinatal infections, such as Zika virus. In addition to antibodies (humoral immunity), clinical tests are now available for cellular immunity in infectious diseases, including interferon-γ release assays tuberculosis and cytomegalovirus infections [36,37].

Serological detection has been recognized for its sensitivity in convalescent patients with COVID-19 and plays an important role for understanding the epidemiology of SARS-CoV-2 and emerging variants, including the burden and role of asymptomatic infections [9]. A meta-analysis of diagnostic performance of the serological tests for COVID-19 revealed the impact of assay design and post-symptom-onset intervals. Using combined nucleocapsid (N) and spike (S) protein had a better sensitivity compared to either N or S protein only. Serological tests played an important role in the clinical diagnosis for the later-stage COVID-19 patients. Enzyme-linked immunosorbent assays (ELISA) for detecting total antibodies or targeting combined N and S proteins had a higher diagnostic sensitivity compared to other methods [38], which have been used successfully for contact tracing in the early COVID-19 pandemic [39]. Lichtenegger et al developed a faster live virus assay to quantitatively detect neutralizing antibodies through the early measurement of antibody-mediated intracellular virus reduction by SARS-CoV-2 real-time PCR [40]. Cell culture-based plaque reduction neutralization assays and their derivatives are the most reliable and accurate methods to determine SARS-CoV-2 neutralization antibody activities [34,35].

The last category of infectious disease diagnostics is molecular biology techniques for the detection of pathogen-specific nucleic acids. PCR, an enzyme-mediated *in vitro* nucleic acid amplification method, has become the dominant method in clinical microbiology services, especially for viral infections [15,22]. A group of simple, rapid and integrated molecular devices is gradually replacing antigen testing for immediate diagnosis at the point of care [41,42]. The “syndromic panel” kits incorporating multiplex-PCR have been widely used to identify a range of pathogens with similar symptoms [42,43]. Quantitative pathogenic testing plays a key role in the monitoring of infection treatment. Next-generation nucleic acid sequencing and gene editing technologies are also finding their way into the detection and identification of specific nucleic acids of pathogenic microorganisms [44,45].

Beyond nucleic acids, matrix-assisted laser desorption ionization-time of flight mass spectrometry (MALDI-TOF MS), which targets microbial proteomic profiles, has been widely used in the clinical microbiology laboratory for rapid and accurate identification [46,47]. In addition, MALDI-TOF MS has been explored for determining epidemic relatedness and antibiotic resistance of microbial isolates. The value of MALDI-TOF MS for microbial typing was investigated in several studies involving *Staphylococcus aureus* [48,49]. Garrigos and colleagues built a database using MALDI-TOF MS allowing rapid and accurate species identification and determination of the multi-resistant epidemic clones of *Achromobacter* species in French cystic fibrosis centers [50]. These data indicated that this technology is a potential rapid screening tool for nosocomial infection investigations. Recently, the MALDI-TOF MS technique has been extended to functional identification including determination of antibiotic resistance [51,52]. Youn et al used the MALDI-TOF MS for the rapid detection of potentially *bla*_KPC_-containing carbapenem-resistant isolates, providing early and clinically actionable results [53]. MALDI-TOF MS profiling in combination with growth media containing isotopically labelled amino acids was reported for the detection of resistant microorganisms after hours of incubation [54]. Scientists in Bruker Daltonik and clinical collaborators reported the rapid detection of antibiotic resistance by MALDI-TOF MS using a direct-on-target microdroplet growth assay [55–57] Detection of colistin resistance became rapid and reliable by use of the MALDI Biotyper Sirius system in both *E. coli* and *P. aeruginosa* [58,59]. These methods being developed based MALDI-TOF MS technology provide an alternative approach to timely monitoring microbial infections [52].

The COVID-19 pandemic ignited the development of numerous nucleic acid amplification methods for the diagnosis and monitoring of SARS-CoV-2 infections. Molecular tests such as real-time PCR are highly sensitive and specific at detecting viral RNA and are now recommended by World Health Organization (WHO) for confirming the diagnosis in individuals who are symptomatic as well as for informing public health decisions. Several newer molecular methods including digital droplet PCR [60] and CRISPR-based assays [61] have been used in COVID-19 diagnostics. Culture accompanied with mNGS increased pathogen diagnostic rate of secondary infections in severe and critical ill COVID-19 patients [62]. Integrated, random-access, point-of-care molecular devices have been developed for fast and accurate diagnosis of SARS-CoV-2 infections in local hospitals and clinics bearing the burden of identifying and treating patients [63,64]. Molecular methods have played significant roles in the discovery and characterization of emerging pathogens such as new world hantavirus [65], influenza A (H7N9) virus [66] and most recently Langya henipavirus [67].

## A bright and evolving future

**Figure 1.**
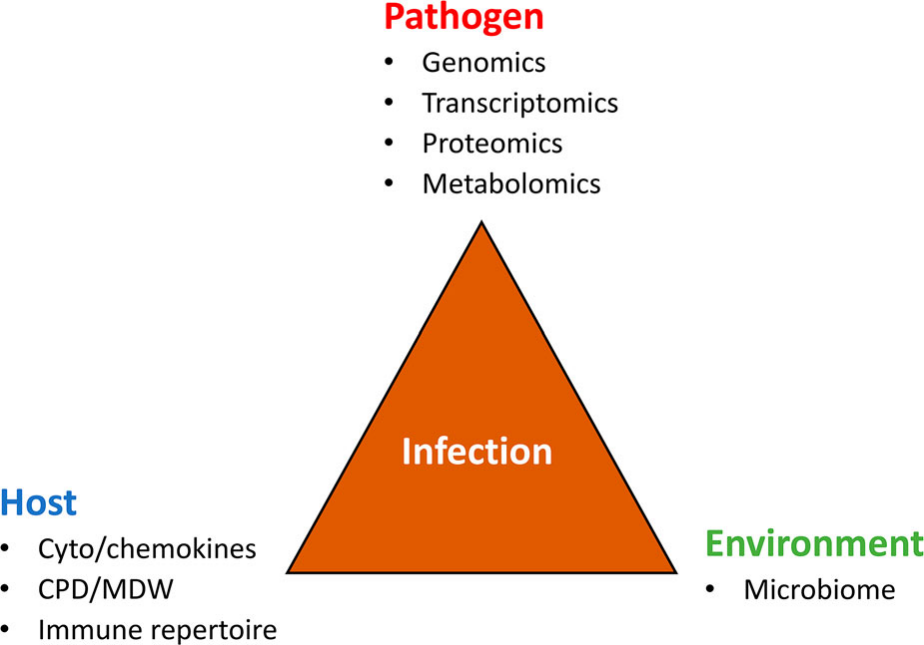
Infection is the result of the interaction between the pathogen, the host and the environment. CPD, cell population data; MDW, monocyte distribution width.

The development and progression of an infectious disease can be thought of as the result of a tripartite interaction between the pathogen, the host, and the environment (Figure 1). On the pathogen side, techniques will continue to evolve to expand and enhance the capacity for detection and characterization of emerging microbial pathogens. Since the genomes of an increasing number of microbial species/strains have been sequenced, the door has been opened wide for “omic” technologies to be used more broadly [68]. Metagenomic next-generation sequencing (mNGS)-enabled surveillance methods offer the opportunity to improve the detection of both known and yet-to-emerge pathogens such as SARS-CoV-2 and the new Langya henipavirus reported recently [67,69,70]. Wilson et al in 2014 reported the use of NGS to provide an actionable diagnosis of neuroleptospirosis [71]. NGS-based system has been commercially available to determine for HIV-1 genotypic resistance directly in clinical samples [72]. The mNGS testing is a powerful tool that can aid in aetiology diagnosis especially in complicated cases as a rule-out. With limitations understood, mNGS data can be useful with host response information incorporated. Currently, turnaround times remain a major hurdle, although same-day result can be produced using faster system such as Nanopore sequencing [73]. Finally, the clinical relevance of the presence of pathogen-specific nucleic acids in a clinical specimen, which may or may not indicate infection, can be adjudicated by a panel of clinical microbiologists and physicians, much like in done in other areas of pathology [74] or a sequencing stewardship panel [75].

Besides genomics, the laboratory may use transcriptomics, proteomics, and metabolomics [76], each of which may carry potential diagnostic utility. For instance, in *Mycobacterium tuberculosis* infections, because the half-life of mRNA is extremely short as compared with rRNA or genomic DNA, assays that target mycobacterial mRNA better reflect mycobacterial viability, which may be used to monitor the efficacy of anti-TB therapy [77]. Regarding metabolomics, Koo et al reported the use of breath fungal secondary metabolite signatures to diagnose invasive aspergillosis infections [78]. On 14 April 2022, the US FDA granted an emergency use authorization for the InspectIR COVID-19 Breathalyzer (InspectIR Systems, Frisco, TX, USA), the first COVID-19 diagnostic test using gas chromatography/gas mass-spectrometry to identify chemical mixtures of five volatile organic compounds (VOCs) associated with SARS-CoV-2 infection in exhaled breath, which provides a SARS-CoV-2 identification result in less than three minutes (https://www.fda.gov/media/157720/download). A series of metabolomic profiles were reported to detect and characterize culturable and unculturable bacterial pathogens in clinical settings [79–81]. In these and numerous other research studies, multi-omic techniques have been leveraged to generate molecular profiles for the surveillance and management of emerging infections [82,83].

Host response markers also have been explored to facilitate diagnosis of microbial infections. Zhang et al. utilized metatranscriptomics of blood from COVID-19 patients and identified a transcriptional signature of differentially expressed genes significantly associated with immune response to SARS-CoV-2 [84]. Furthermore, Sweeney et al. conducted integrated multi-analyte profiling to yield a three-gene set for testing whole blood specimens that is very robust for diagnosing active tuberculosis (with potential relevance both for diagnosis and treatment monitoring) [82]. Cepheid (Sunnyvale, CA, USA) has recently developed a prototype assay (Xpert MTB® MTB Finger Stick) to detect a three gene host response signature in whole blood specimens using the GeneXpert® system. The gene signature may be of value both for diagnosis and treatment monitoring. Several studies have demonstrated that the assay fulfils the most of the attributes of the WHO target product profile for a point of care triage test for TB. The assay can be performed on fingerstick blood [85–88]. A novel assay called MeMed BV that integrates measurements of blood-borne host-proteins (tumour necrosis factor-related apoptosis-inducing ligand, interferon γ-induced protein-10, and CRP) was developed and manufactured by MeMed Diagnostics (Tirat Carmel, Israel) to assist in differentiation between bacterial and viral disease [83,89]. A recent prospective, multicenter cohort study performed by Papan et al. validated the high diagnostic performance of the MeMed BV assay in a broad paediatric cohort, and supported its potential to reduce antibiotic overuse in children with viral infections [90].

It is now well-established that the gut microbiota plays a critical role in infection pathogenesis [91,92]. The importance of the human microbiome is becoming increasingly evident. While the contributions of individual microbes in the human health are still far from fully understood, the microbiome appears to play a key role in many vital functions, including synthesizing vitamins and amino acids, generating important metabolites, protecting against pathogens, utilizing non-human biochemical pathways and contributing to the immune system. A large body of evidence demonstrated, more than a decade ago, that gut microbial alteration is a key factor in the pathogenesis of many local and systemic disorders, including infections [93]. Characterization of the composition of the gut microbiota as well as a dominant pathogen(s) in patients with microbial infections promises to open new avenues for the development of patient-centered personalized and precision diagnosis [94]. One example was to used robust microbiota-based assay to enable simple diagnostics and disease activity monitoring for inflammatory bowel disease [95]. Laboratory-developed tests are commercially available for microbiome determination in several reference laboratories. For example, the Gut Intelligence Test (Viome Inc., Los Alamos, NM, USA) uses a robust and automated stool metatranscriptomic method oﬀering a rapid and comprehensive taxonomic and functional readout of the gut microbiome [96]. An integrated diagnostic approach combining pathogen, host and microbiome would enhance the speed and accuracy for the laboratory diagnosis and monitoring of microbial infections (Figure 1). Among them, machine learning will gradually apply in clinical microbiology practice especially for unusual emerging pathogens including predicting drug targets or vaccine candidates, diagnosing microorganisms causing epidemics, predicting disease outbreaks and exploring microbial interactions [97–99]. Machine learning has been used in the clinical setting for classifying drug resistance against antimicrobial agents [100,101].

**Figure 2.**
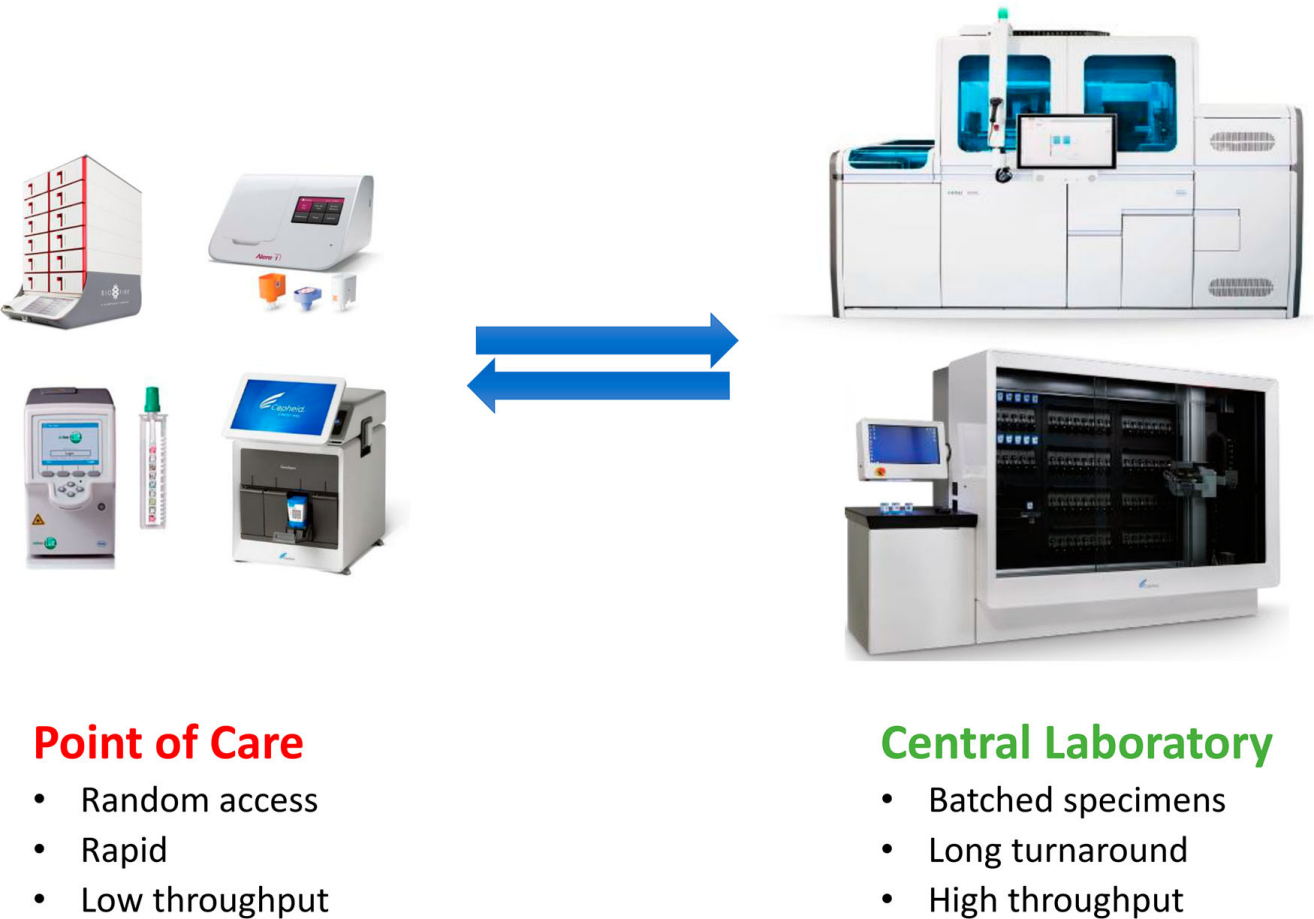
Polarized clinical microbiology practice in the near future with rapid, random-access tests done at point of care (left) and with batched, large volumes of tests done at central laboratory (right).

In the future, there will be a need for more rapid diagnoses, increased standardization of testing and greater adaptability to cope with new threats from emerging microbial pathogens. As early as 2004, Didier Raoult, the renowned French medical and clinical microbiologist, jointly with his team, proposed a bipolarization of future clinical microbiology services [102] (Figure 2). On one side, clinical microbiology practice will follow the general trend in the life sciences for large, centralized laboratories with the capacity to analyze large numbers of samples and to carry out a wide range of techniques. Total automation has been gradually achieved for bacterial culture, identification and antimicrobial susceptibility testing [23]. For molecular diagnosis, molecular platforms are increasingly designed with an emphasis on automation and sample-to-result capabilities. These include technologies by Roche (4800/6800/8800 platforms) [103], Abbott Laboratories (m2000 and Alinity m) [104], Hologic (Panther and Panther-Fusion) [105], Becton Dickinson (BDMax and BD COR) [106], and Cepheid (GeneXpert Infinity) [107].

On the other side, rapid and on-demand testing is performed at point of care (POC) based with relatively low throughput testing volumes. These include rapid screening for influenza in the emergency room and rapid mixed-sample screening for new coronaviruses in the field [41,108]. The current widely accepted definition of POC testing includes testing that occurs at or near the point of patient care, such that the results drive patient care decisions made during that encounter [109,110]. POC tests can be performed in a variety of settings including physician offices, emergency department, urgent care facilities, school health clinics and pharmacies. Recently, the COVID-19 pandemic has shined a spotlight on Clinical and Laboratory Improvement Amendments (CLIA)-waived diagnostic testing. Some SARS-CoV-2 tests have received Emergency Use Authorization (EUA) from the U.S. Food and Drug Administration (FDA) for use in CLIA-waived testing sites [108]. Further optimization and validation, new technologies, as well as studies to determine clinical and epidemiological impact of SARS-CoV-2 POC tests are needed. Nevertheless, random-access, integrated devices available at the point of care with scalable capacities will increase its weight in the rapid and accurate diagnosis and monitoring of emerging pathogens in the near future.

In summary, the discipline of clinical microbiology, with a long and uneven past, has been thriving at present and is ready to embrace the future. There have been substantial changes in the role of clinical microbiology laboratories over the past decade. The ongoing technological revolution has rapidly transformed research, diagnostic and therapeutic tools. In the near future, clinical microbiology practice will be able to help clinicians implement real-time evidence-based treatments or significantly shorten the process from empirical to evidence-based treatments. While continuously strengthening its scientific attribution, clinical microbiology will be endowed with a more distinct, more profound, more ambitious medical landscape, social value and management significance. Full of challenges and uncertainties, though, the practice of clinical microbiology will keep abreast of the times, promising and never fading, for its demand-oriented, significance-driven, logic and science-based, and humanitarian characteristics.
